# Self-Treatment

**Published:** 1906-01

**Authors:** Claude B. Warner


SELF-TREATMENT.—The country dentist may often save himself and his patients much trouble by ordering a gross of one drachm vials with corks. By giving the patient medicine and letting him make treatment himself at regular intervals or when pain prompts, much suffering can be avoided. Beeswax is a convenient sealing material in such a case. Of course, the dentist must give the later treatment himself.—*Dr. Claude B. Warner, Tri-State Dental Quarterly.*

